# Cancer and Cardiovascular Disease: The Complex Labyrinth

**DOI:** 10.1155/2015/516450

**Published:** 2015-08-04

**Authors:** Susan Dent, Peter Liu, Christine Brezden-Masley, Daniel Lenihan

**Affiliations:** ^1^The Ottawa Hospital Cancer Centre, Department of Medicine, University of Ottawa, Ottawa, ON, Canada K1H 8L6; ^2^University of Ottawa Heart Institute, Department of Medicine, University of Ottawa, Ottawa, ON, Canada K1Y 4W7; ^3^Division of Hematology/Oncology, St. Michael's Hospital, Toronto, ON, Canada M5B 1W8; ^4^Division of Cardiology, Vanderbilt University Medical Centre, Nashville, TN 37232, USA

As our population ages, there has been an increase in the prevalence of cancer and heart disease [1]. Modern treatment strategies have led to improvement in the chances of surviving a diagnosis of cancer; however, these treatments can come at a cost [2]. Cardiotoxicity, a relatively new term in the medical literature, refers to the impact of cancer therapies on the heart and cardiovascular system [3, 4]. Cohort studies in pediatric cancer survivors have shown that cardiotoxicity is the second leading cause (after cancer recurrence) of morbidity and mortality in cancer survivors [5]. The potential negative impact of cancer drugs on the heart, however, is not new. In fact, we have known for years that cancer drugs, such as the anthracyclines, can cause severe and permanent heart damage including heart failure (HF). So why is there growing interest now?

In 2005 trastuzumab in combination with chemotherapy was shown to significantly improve disease-free and overall survival in women with early stage HER2 positive breast cancer [6, 7]. While the dramatic improvements in clinical outcomes led to the widespread adoption of this treatment in clinical practice, it became readily apparent that women were experiencing higher rates of cardiac dysfunction than had been anticipated during clinical development—thus placing oncologists in a difficult situation—to treat or not to treat [8]!

The last several years have seen the development and approval of a plethora of cancer drugs, many of which may negatively impact the heart and cardiovascular system. Tyrosine kinase inhibitors (e.g., sunitinib) can cause or exacerbate preexisting hypertension and BCR-ABL inhibitors (e.g., dasatinib) can cause Q-T prolongation. In the modern era of cancer therapy it is imperative that oncologists work closely with cardiologists in order to provide the best possible cancer care without compromising cardiac health [9]. This is particularly important for those patients with preexisting heart disease who then develop cancer and are exposed to potentially cardiotoxic cancer drugs.

In this special issue we gain insight into the challenges that health care providers face when treating this unique population of patients. While our understanding of how modern cancer therapies impact the heart continues to evolve, many knowledge gaps persist.

How do we identify cancer patients at high risk of cardiotoxicity? In this issue, M. Davis and colleagues highlight the importance of cardiovascular risk assessment in cancer patients prior to commencing therapy. In a cohort of prostate cancer patients, they identified a high prevalence of baseline cardiovascular risk factors and cardiovascular disease (25%) prior to initiation of cancer therapy. A standardized approach of cardiovascular risk assessment prior to initiation of treatment is needed for all cancer patients in order to optimize cardiovascular health prior to, during, and after treatment.

What are the best modalities to detect cardiotoxicity? Two-dimensional (2D) echocardiography and MUGA scans are the most widely used modalities for monitoring cardiac function in chemotherapy treated patients—but is this the best strategy? F. Pizzino and colleagues discuss newer imaging modalities, including 2DE tissue Doppler imaging (TDI), cardiac magnetic resonance imaging (CMR), and 2D and 3D speckle tracking echocardiography. Left ventricular ejection fraction (LVEF) has been the “gold standard” used to detect cardiotoxicity—but it is clear that this is not the best method [10]. A. Calleja and colleagues evaluated right ventricular (RV) function in breast cancer patients receiving trastuzumab (+/−anthracyclines) who had left ventricular defined cardiotoxicity. Patients with RV dysfunction at the time of LV-related cardiotoxicity had reduced recovery of LVEF although this was not statistically significant. Further research is clearly needed to determine which imaging modalities will provide the most accurate and reproducible information to detect “early” cardiotoxicity and what parameters we should be measuring in order to facilitate early intervention strategies.

How do we manage cardiotoxicity in this patient population? In this issue, J. Sulpher and colleagues clearly identify knowledge gaps between cardiologists and oncologists in the appropriate clinical management of cancer patients who develop cardiotoxicity secondary to their cancer treatment, underscoring the need for collaboration between oncologists and cardiologists. In order to facilitate this collaboration, a number of dedicated cardiac oncology clinics have been established (mainly in academic centers) but are these specialized clinics impacting patient care? J. Sulpher and colleagues describe the clinical outcomes of cancer patients referred to a dedicated cardiac oncology clinic. While their conclusions are limited by the observational nature of their study, their results are encouraging (majority of cancer patients completed treatment) and support ongoing collaboration and research in this area.

And finally how do we manage those patients who develop end stage heart disease due to cancer therapy? N. Ghosh and colleagues describe the unique challenges and clinical outcomes of cancer patients with end stage heart failure who require advanced therapies such as inotropic support, orthotopic heart transplantation, or left ventricular assist devices.

Modern cancer therapies have led to more individuals surviving a diagnosis of cancer. There is an increasing appreciation, by health care providers, of the potential negative impact of cancer therapies on cardiovascular health. This special issue adds to our current knowledge in the discipline of cardiac oncology and we look forward to future research that will help guide best practices.

*Susan Dent* *Susan Dent* *Peter Liu* *Peter Liu* *Christine Brezden-Masley* *Christine Brezden-Masley* *Daniel Lenihan* *Daniel Lenihan*
